# Biodegradation of Di-(2-ethylhexyl) Phthalate by *Rhodococcus ruber* YC-YT1 in Contaminated Water and Soil

**DOI:** 10.3390/ijerph15050964

**Published:** 2018-05-11

**Authors:** Ting Yang, Lei Ren, Yang Jia, Shuanghu Fan, Junhuan Wang, Jiayi Wang, Ruth Nahurira, Haisheng Wang, Yanchun Yan

**Affiliations:** 1 Graduate School, Chinese Academy of Agricultural Sciences, Beijing 100081, China; 13838348327@163.com (T.Y.); jia_yang@outlook.com (Y.J.); fanshuanghu@126.com (S.F.); wangjunhuan_1993@163.com (J.W.); 15524115399@163.com (J.W.); ruth.nahurira@yahoo.com (R.N.); wanghaisheng@caas.cn (H.W.); 2 Agricultural College of Guangdong Ocean University, Zhanjiang 524088, China; lren_87@hotmail.com

**Keywords:** biodegradation, di-(2-ethylhexyl) phthalate, *Rhodococcus ruber*, marine plastic debris, bioremediation

## Abstract

Di-(2-ethylehxyl) phthalate (DEHP) is one of the most broadly representative phthalic acid esters (PAEs) used as a plasticizer in polyvinyl chloride (PVC) production, and is considered to be an endocrine-disrupting chemical. DEHP and its monoester metabolites are responsible for adverse effects on human health. An efficient DEHP-degrading bacterial strain *Rhodococcus ruber* YC-YT1, with super salt tolerance (0–12% NaCl), is the first DEHP-degrader isolated from marine plastic debris found in coastal saline seawater. Strain YC-YT1 completely degraded 100 mg/L DEHP within three days (pH 7.0, 30 °C). According to high-performance liquid chromatography–mass spectrometry (HPLC-MS) analysis, DEHP was transformed by strain YC-YT1 into phthalate (PA) via mono (2-ethylehxyl) phthalate (MEHP), then PA was used for cell growth. Furthermore, YC-YT1 metabolized initial concentrations of DEHP ranging from 0.5 to 1000 mg/L. Especially, YC-YT1 degraded up to 60% of the 0.5 mg/L initial DEHP concentration. Moreover, compared with previous reports, strain YC-YT1 had the largest substrate spectrum, degrading up to 13 kinds of PAEs as well as diphenyl, p-nitrophenol, PA, benzoic acid, phenol, protocatechuic acid, salicylic acid, catechol, and 1,2,3,3-tetrachlorobenzene. The excellent environmental adaptability of strain YC-YT1 contributed to its ability to adjust its cell surface hydrophobicity (CSH) so that 79.7–95.9% of DEHP-contaminated agricultural soil, river water, coastal sediment, and coastal seawater were remedied. These results demonstrate that *R. ruber* YC-YT1 has vast potential to bioremediate various DEHP-contaminated environments, especially in saline environments.

## 1. Introduction

Phthalic acid esters (PAEs) are a family of refractory organic compounds that are widely used as plasticizers for polyvinyl chloride (PVC) and other polymers to increase flexibility and longevity [1,2]. PAEs are also used as additives in personal care products and medical devices [3]. PAEs easily leach into the environment, mainly from domestic and industrial effluents, plastic decomposition, and agricultural or urban outflow [4]. In addition, PAEs are classified as endocrine-disrupting chemicals (EDCs) due to their xenoestrogenic and endocrine-disrupting characteristics [4,5,6]. Di-(2-ethylhexyl)-phthalate (DEHP) is the most widely-used phthalate, and is listed as a priority hazardous substance by the European Community, the United States Environmental Protection Agency, and the China National Environmental Monitoring Center [7,8]. Previous studies showed that DEHP and its dominant metabolites, mono (2-ethylehxyl) phthalate (MEHP) and phthalic acid (PA), the major intermediate degradation products of most PAEs (including DEHP), produce adverse developmental, neurological, respiratory, and immune effects in humans [4,9,10,11,12,13]. Therefore, discovering efficient DEHP degraders with high environmental persistence and wide availability is urgently required, as is establishing remediation strategies to eliminate DEHP and its metabolites from the environment.

In nature, DEHP is not readily removed by hydrolysis or photolysis [11,14,15,16]. Microbial degradation is the most effective and promising means of removing DEHP from aquatic and terrestrial systems, and is the most promising process for remediating contaminated environments [10,17,18,19,20,21]. Many microorganisms with DEHP-degrading abilities and their characteristics are briefly summarized in Table 1. Among these, various genera such as *Rhodococcus* [7], *Gordonia* [22], *Pseudomonas* [23], *Sphingomonas* [24], *Arthrobacter* [25], *Achromobacter* [26], *Bacillus* [21], *Providencia* [11], *Acinetobacter* [27], and *Microbacterium* [28] have been isolated from activated sludge, river sediment, wetland, heavily plastics-contaminated sewage, aerobic granules, compost-amended soil, vegetable greenhouse soil, and municipal solid waste. Although the DEHP-degraders were isolated from various environments, no studies have examined the degradation of DEHP by strains inhabiting coastal saline environments, despite the fact that accumulated marine plastic debris heavily pollutes this ecosystem. Exploring marine bacteria that can remove DEHP in salty environments could be a meaningful research focus. Several kinds of PAEs or polycyclic aromatic hydrocarbons (PAHs) often simultaneously exist in the natural environment [4,10,29]. However, in most cases, even the DEHP-degraders with the widest substrate spectrum can degrade no more than eight kinds of PAEs. For instance, *Pseudomonas fluorescens* FS1 [30], *Bacillus megaterium* YJB3 [2], *Providencia* sp. 2D [11], *Gordonia alkanivorans* YC-RL2 [22], *Bacillus subtilis* No.66 [21], and *Mycobacterium* sp. YC-RL4 [28] can degrade five kinds of PAEs. Strains *Gordonia* sp. Dop5 [20], *Rhodococcus* sp. HS-D2 [31], and *Sphigomonas* sp. DK4 in conjunction with *Corynebacterium* sp. O18 [24] could degrade eight kinds of PAEs. Thus, finding one strain that has the ability to function in complex environments is important for remediating DEHP-contaminated environments. Attention should also be paid to the degradation of lower concentrations of DEHP [10,32]. The no-observed-effect concentrations (NOECs) of DEHP are 77 μg/L in surface water [33,34] and 470 μg/kg dry weight in sediment (Sediment Management Standards, 1991), and the environmental risk limit (ERL) for DEHP in soil is 1000 μg/kg fresh weight [35]. Lower concentrations of DEHP not only cause serious health problems in humans [12,36,37], but are also faced with degradation difficulties for certain strains due to the low bioavailability of pollutants [10]. In summary, some other characteristics of the degraders should be confirmed, and more efforts should be made to promote the bioremediation of DEHP-contaminated environments.

Based upon the background above, the present study concentrated on the degradation of DEHP by a marine bacterium with super salt tolerance belonging to the genus *Rhodococcus*. The environmental factors (pH, temperature, and salinity) were investigated to study the degradation characteristics. The substrate range and the maximum and minimum degrading capacity were also evaluated. DEHP metabolites were detected, and a possible biodegradation pathway was deduced. To assess the application potential of the strain for the bioremediation of DEHP-contaminated environments, we measured the variation in cell surface hydrophobicity (CSH) and estimated the efficiency during DEHP degradation.

## 2. Materials and Methods

### 2.1. Chemicals

DEHP, dimethyl phthalate (DMP), diethyl phthalate (DEP), di-*n*-butyl phthalate (DBP), benzyl butyl phthalate (BBP), di-cyclohexyl phthalate (DCHP), di-propyl phthalate (DPrP), dipentyl phthalate (DAP), dihexyl phthalate (DHP), di-*n*-heptyl phthalate (DHPP), dioctyl phthalate (DOP), di-nonyl phthalate (DNP), and di-decyl phthalate (DDP) (all with purity above 99%), diphenyl, p-nitrophenol (PNP), PA, benzoic acid (BA), phenol, protocatechuic acid (PCA), salicylic acid (SA), catechol, and 1,2,3,4-tetrachlorobenzene were purchased from Sinopharm Chemical Reagent Co., Ltd. (Beijing, China). PAEs stock solution was 20,000 mg/L in methanol. All other chemicals and solvents used were of analytical and high-performance liquid chromatography (HPLC) grade, respectively.

### 2.2. Domestication, Selection, and Identification of DEHP-Degrading Strain

Marine plastics debris sludge and water samples were collected from the Dameisha coastline, Nanshan district, Shenzhen, China, located at north latitude 22.59° and east longitude 114.31°. The sludge and water sample (10 g, wet weight) was mixed with 100 mL of a Basal Medium (BM: MgSO_4_·7H_2_O 0.2 g/L, FeSO_4_·7H_2_O 0.001 g/L, KH_2_PO_4_ 1.5 g/L, (NH_4_)_2_SO_4_ 2.0 g/L, CaCl_2_ 0.001 g/L, Na_2_HPO_4_·12H_2_O 1.5 g/L in double distilled water, pH 7.0) with 100 mg/L DEHP and was incubated at 30 °C for 7 days with shaking at 180 r/min. After acclimation for six rounds, the concentration of total DEHP was increased gradually from 100 to 600 mg/L in the BM medium. Luria-Bertani plate medium (peptone 10.0 g/L, yeast extract 5.0 g/L, NaCl 10.0 g/L, and agar 15 g/L) was used for enrichment. The plates were incubated at 30 °C and the potential DEHP-degrading bacteria were purified by using the plate-streaking technique. Each possible single colony was collected, and the degrading capability was verified in BM containing 100 mg/L DEHP. Culture without inoculation containing 100 mg/L DEHP was set as the abiotic control. The residual concentration of DEHP was detected with a gas chromatograph (GC). All experiments were performed in triplicate.

The morphology of the YC-YT1 strain was investigated by scanning electron microscope [48] (SEM, Hitachi-SU8010). The physio-biochemical characteristics were examined with the BIOLOG Micro-station (BIOLOG Inc., Hayward, CA, USA). Strain YC-YT1 was deposited in the China General Microbiological Culture Collection Center (CGMCC) under the accession number **CGMCC 13959**. A bacterial DNA kit (OMEGA BioTek, Norcross, GA, USA) was used for the total genomic DNA extraction. The universal primers 27F (5′-AGAGTTTGATCCTGGCTCAG-3′) and 1492R (5′-GGTTACCTTGTTACGACTT-3′) were used for amplification of the 16S rRNA gene. The purification and sequencing of 16S rRNA was conducted by Sangon Biotech (Shanghai, China). The resulting sequence was submitted to GenBank (**KY228387**) and compared with known sequences using the basic local alignment search tool (BLAST). A phylogenetic tree was constructed by the neighbor-joining algorithm using MEGA 6.0 [49]. BIOLOG MicroStation is a standardized micro-method which was used to determine the use of the carbon source [28].

### 2.3. Biodegradation Batch Experiment of DEHP by Strain YC-YT1

To characterize the ability of YC-YT1 to degrade DEHP, a series of batch experiments were conducted. In the following investigations, the strain YC-YT1 was grown to an OD_600_ of 0.6 and harvested by centrifugation (4000 r/min, 5 min), washed three times with phosphate buffer solution (PBS) and resuspended in BM (approximately 6.5 × 10^7^ cells/mL). The inoculation proportion of the seed was 1.0% of the culture (*v*/*v*), unless stated otherwise. The medium without inoculation of strain YC-YT1 served as the control.

DEHP biodegradation by strain YC-YT1 was affected by different environmental factors, such as pH, temperature, salinity, and glucose concentration. To determine the optimal conditions for DEHP degradation by strain YC-YT1, single-factor optimization experiments were performed in this study, including pH (4.0, 5.0, 6.0, 7.0, 8.0, 9.0, and 10.0), temperature (10, 20, 30,40, and 50 °C), salinity (1, 3, 5, 7, 10, 15, 20, 25, 40, 50, 60, 70, 80, 90, 100, 110, and 120 g/L), and glucose concentration (0, 2.5, 5, 7.5, and 10 g/L). We set the initial concentration of DEHP to 100 mg/L in the optimization tests. The control was used as stated above without inoculation of the seeds. All cultures were incubated in a shaker (180 r/min) at 30 °C. All experiments were conducted in triplicate and the residual DEHP concentration was measured by GC.

### 2.4. Efficient Degradation of DEHP at Maximum and Minimum Concentrations

In natural environments, the concentration of DEHP is very low. Thus, it is important for the strain to be able to have degradation ability at low DEHP concentrations. Conversely, the strain’s bioremediation application ability against high concentration pollutants must be determined. Maximum and minimum concentration tests were performed with different initial concentrations of DEHP (max, 50, 100, 200, 300, 400, 500, 600, 700, 800, 900, and 1000 mg/L; min, 0.5, 1, 2, 5, and 10 mg/L). After incubating for 3 days, the DEHP concentration was analyzed using GC, and the degradation rate was calculated.

### 2.5. Substrate Use Tests

Strain YC-YT1 was tested for its ability to grow on various PAEs and some organic compounds. The isolated YC-YT1 was cultured in 20 mL BM containing one (100 mg/L) of the following substrates as the sole source of carbon and energy: DMP, DEP, DBP, DPrP, DHPP, DNP, DOP, DEHP, DAP, BBP, DDP, DHP, DCHP, diphenyl, PNP, PA, BA, phenol, PCA, SA, catechol, and 1,2,3,4-tetrachlorobenzene. Cultures were replicated three times and incubated at 30 °C, 180 r/min for 3 days. Non-inoculated cultures served as the control. The structures of the target contaminant substrates are presented in Supplementary file.

### 2.6. Bioremediation of DEHP-Contaminated Environments and Evaluation the Cell Surface Hydrophobicity

The DEHP degradation efficiency of strain YC-YT1 was measured in real natural samples. Soil (garden and wheat field soil) and water (pond water) samples were gathered from an agricultural field and a garden of the Chinese Academy of Agricultural Sciences (Beijing, China). We also collected coastal seawater and sediment samples in the intertidal zone from the Dameisha coastline in March 2018. Sediment samples (0–5 cm, Supplementary Figure S4) with the upper seawater layer were collected in triplicate using a sterile shovel. The initial DEHP concentrations were examined to eliminate the inherent effects of DEHP. The recorded physico-chemical characteristics of samples are listed as follows. Soil (dry weight): total N, total P, organic matter, and pH; pond water: suspended solids (SS), chemical oxygen demand (COD), conductivity, and pH; sediment: pH, salinity, total organic carbon, total N, total P, moisture content, and oxidation–reduction potential; seawater: pH, salinity, optical density, conductivity, COD, and SS. Detailed characteristics of the samples are described in Supplementary Table S1.

A total of 100 g of the soil (sieved < 0.35 mm) and the coastal sediment as well as pond water and coastal seawater were placed in 500 mL round flask/250 mL triangular flask and supplemented with DEHP (100 mg/kg or mg/L) in methanol solution. Before adding the YC-YT1 suspension into the sample (in triplicate). The suspension was thoroughly mixed to reach a terminal concentration of approximately 1 × 10^7^ cells/mL using drip irrigation technique. The sterile and non-sterile treatments (in triplicate) without YC-YT1 were added as negative controls. All treatments were incubated at a constant temperature of 30 °C and a 60% water-holding capacity was maintained using an artificial light incubator (Table 2). After 7 days of cultivation, residual DEHP concentration was measured in samples (20 g). Samples extraction procedures and the calculation equations of the DEHP degradation rate were conducted as previously reported by our laboratory [28].

Five treatments were designed to measure the variation in CSH: 5 g/L glucose and 50, 100, 200, and 400 mg/L of DEHP. The modified microbial adherence to hydrocarbon (MATH) method was used to evaluate the CSH of the YC-YT1 strain, and the calculation equations were obtained from Ren [28].

### 2.7. Analysis of Chemicals and Metabolites

The concentrations of DEHP and other PAE substrates were analyzed using GC (GC-2010 SHIMADZU, Kyoto, Japan), and the standard curves were constructed (all *R*^2^ above 0.99). The filtrate extraction and the detection parameters were conducted as previously reported [24,31]. Briefly, the cultures were extracted using equal volumes of *n*-hexane twice, and organic phases were centrifugated at 10,000 r/min for 5 min. The exaction efficiency was measured (all above 98%).

The residual DEHP and its degradation intermediates degraded by YC-YT1 were analyzed by HPLC–mass spectrometry (HPLC-MS). The filtrate extraction and cleanup procedures were conducted as per Lin et al. [50]. In brief, the cultures were extracted using ethyl acetate, and then the organic phases were separated by stratification for 2 h. The aqueous phase was extracted twice, after purification and drying by N_2_ and the residue was dissolved in methanol. All the experiments were performed in triplicate. Eventually, the extracts were filtered by a 0.22-μm membrane and 1.0-μL filtrates were injected into HPLC-MS. The detection parameters and experimental procedures were conducted as previously reported [22,28].

## 3. Results

### 3.1. Isolation and Identification of DEHP-Degrading Bacterium

**Figure 1:**
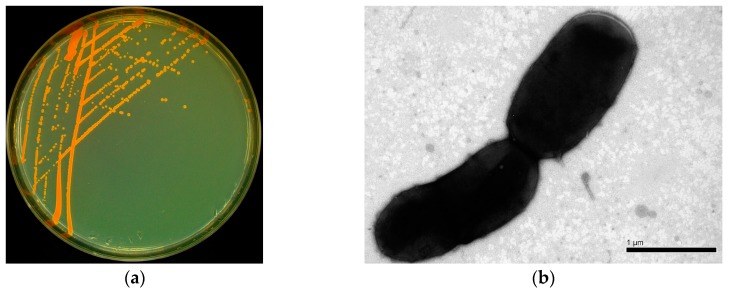
(**a**) The colonial morphology of strain YC-YT1 on a Luria-Bertani plate; (**b**) Morphological characteristics of strain YC-YT1 by scanning electron microscope (Hitachi-SU8010).

**Figure 2:**
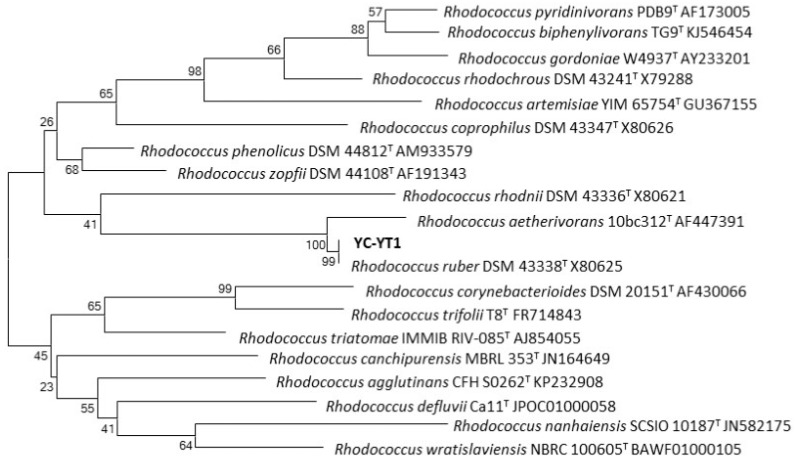
Phylogenetic analysis of strain YC-YT1 based on 16S rRNA gene sequence analysis. The scale bar equals 0.02 changes per nucleotide position.

A DEHP-degrading bacterium YC-YT1 was isolated from marine plastic debris in coastal seawater by enrichment and domestication. It is a Gram-positive short-rod bacteria, the colonies on the Luria-Bertani plates were smooth, lustrous, and orange (Figure 1a), and the cells were rod-shaped under the SEM (Figure 1b). The phylogenetic tree demonstrated that strain YC-YT1 was classified as a *Rhodococcus* species and had a 99% similarity to *Rhodococcus ruber* DSM 43338 (GenBank accession number X80625) based on 16S rRNA gene analysis (Figure 2). The BIOLOG tests were successfully completed (Probability = 0.596, Similarity = 0.596, and Distance = 5.854) and consistent with 16S rRNA analysis. Due to its morphological characteristics and 16S rRNA gene sequence analysis, the YC-YT1 strain was identified as *Rhodococcus ruber*.

### 3.2. Effects of Environmental Factors on DEHP Degradation

#### 3.2.1. Effect of pH on Degradation of DEHP

**Figure 3:**
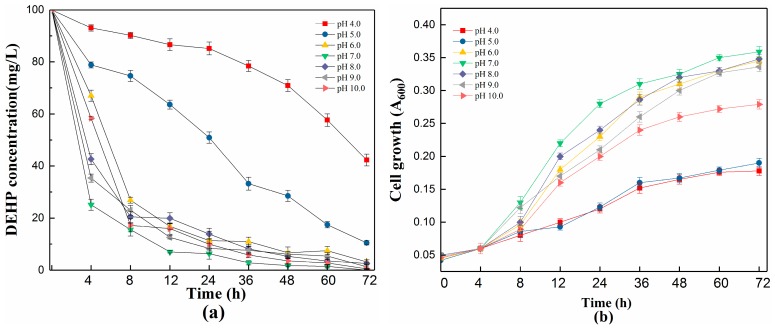
Effects of initial pH on DEHP degradation and cell growth of strain YC-YT1. (**a**) Effects of initial pH on DEHP degradation; (**b**) Effects of initial pH on the cell growth of strain YC-YT1. Error bars indicate standard deviations of the means.

Environmental factors such as pH, temperature, salinity, and glucose concentration play an important role in affecting cell growth, the efficiency of biodegradation, and the enzymatic activation involved in these metabolic pathways [50,51]. As shown in Figure 3, strain YC-YT1 showed different degradation rates at pH values under ranging from 4.0 to 10.0. The degradation efficiency was above 89.5% at pH 5.0–10.0, and 100 mg/L DEHP was completely removed after 72 h incubation under pH 7.0. Compared with acid solution, strain YC-YT1 degraded faster in alkaline solutions, meaning alkaline conditions are more suitable for DEHP degradation. The degradation efficiencies under different acidity–alkalinity conditions were as follows: pH 4.0 (57.7%), pH 5.0 (89.5%), pH 6.0 (96.8%), 7.0 (100%), 8.0 (97.4%), 9.0 (98.7%), and 10.0 (99.5%). Thus, the DEHP degradation efficiencies from pH 6.0 to 10.0, which were better than pH 4.0 to 5.0, suggested that the optimum pH range for DEHP degradation by YC-YT1 is 6.0 to 10.0. YC-YT1 cell density was associated with a concomitant increase in the process of removing DEHP. Despite this, the cell growth at pH 5.0 was obviously decreased, but it was not completely inhibited.

#### 3.2.2. Effect of Temperature on DEHP Degradation

**Figure 4:**
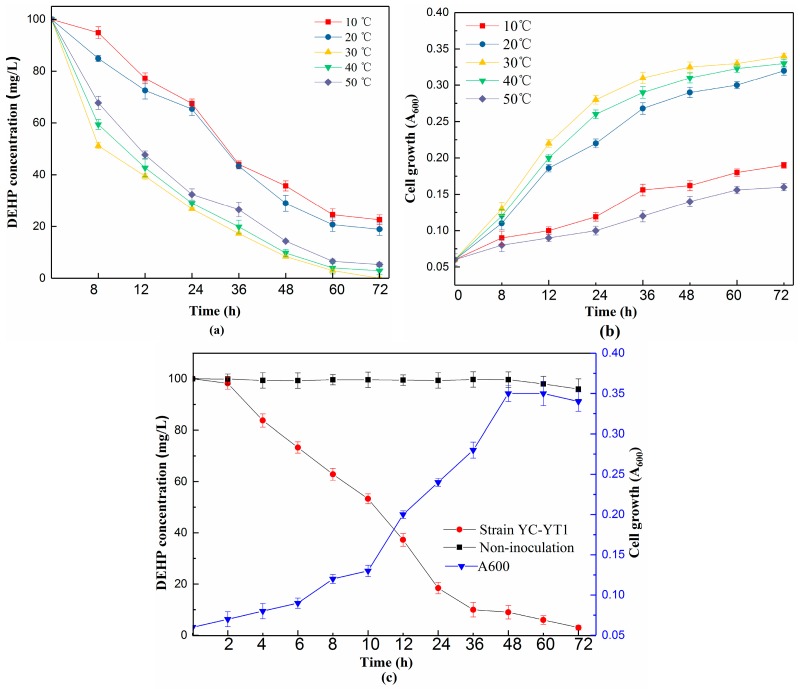
Effects of incubation temperature on the degradation of DEHP and cell growth of strain YC-YT1. (**a**) Effects of incubation temperature on the degradation of DEHP; (**b**) Effects of incubation temperature on the cell growth of strain YC-YT1; and (**c**) DEHP degradation rate and cell growth of strain YC-YT1 under optimized conditions. Error bars indicate standard deviations of the means.

The DEHP degradation efficiency by YC-YT1 was measured at temperatures ranging from 10 to 50 °C. As shown in Figure 4, the DEHP degradation rates were increased from below 80% (≤20 °C) to above 96% (≥30 °C) within 72 h. The optimal temperature was around 30 °C. Lower temperatures (≤20 °C) were less favorable for DEHP degradation. Under the optimized conditions (pH 7.0, 30 °C, and 100 mg/L of DEHP), the lag phase shortened to 10 h cultivation (Figure 4c), and the DEHP degradation rate of YC-YT1 increased to approximately 50%, then completely degraded after 72 h.

#### 3.2.3. Effect of NaCl and Glucose Concentration on DEHP Degradation

**Figure 5:**
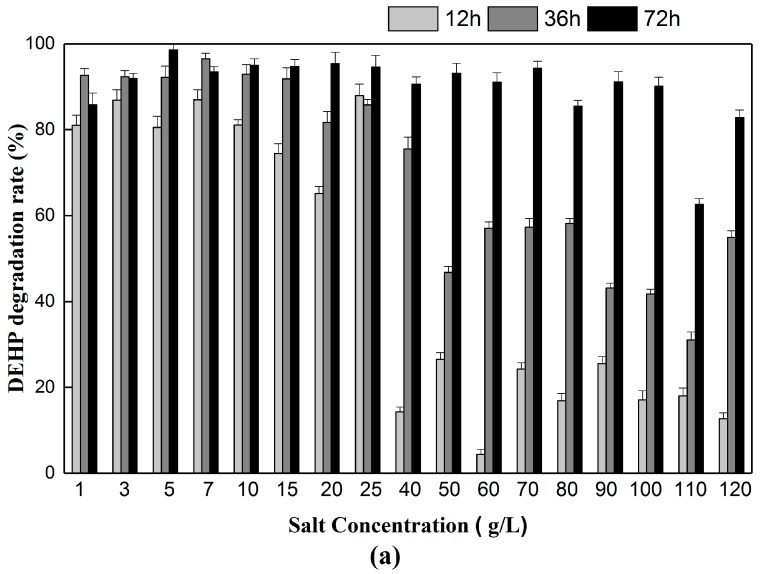

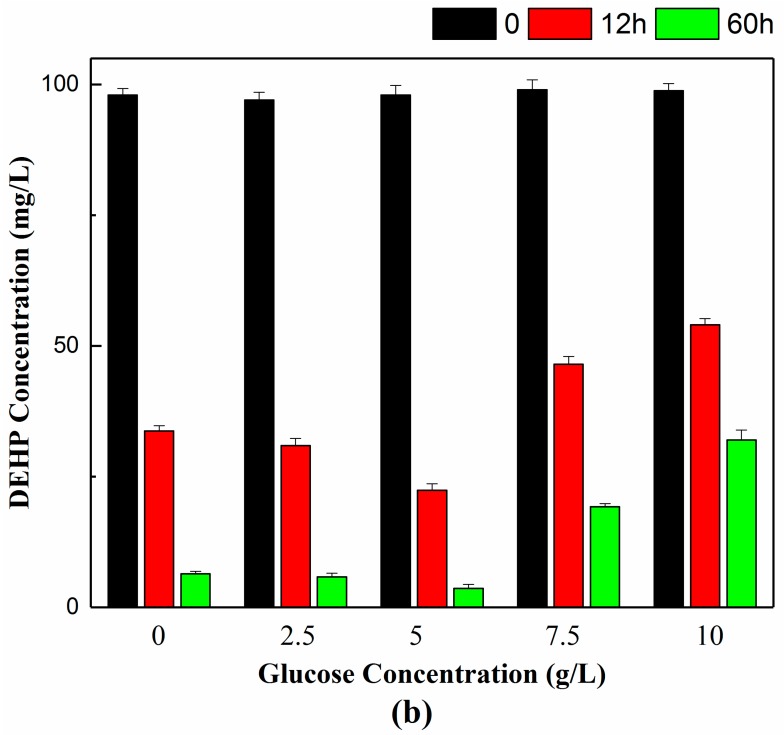
(**a**) Effects of sodium chloride (NaCl) concentration on DEHP degradation. DEHP degradation efficiency by strain YC-YT1 under different NaCl concentrations. (**b**) Effects of glucose concentration on DEHP degradation. DEHP degradation efficiency by strain YC-YT1 under different glucose concentrations. Error bars indicate standard deviations of the means.

Industrial wastewater has high salinity, creating a considerable obstacle for bioremediation [12]. In our study, the effect of salt concentration on DEHP biodegradation was also investigated. After 72 h of cultivation, strain YC-YT1 could bear a maximum NaCl concentration of up to 120 g/L, at which point the degradation rate was above 80% (Figure 5a). The results show that the YC-YT1 strain has the potential to treat wastewater without desalinization. Moreover, adding glucose has been a common approach to study all deviation of resource limitations [52,53]. We also investigated the effect of glucose concentration on DEHP biodegradation after 72 h cultivation, at concentrations of glucose (5 g/L) where YC-YT1 strain had the optimum degradation efficiency (Figure 5b). The results may be due to strain YC-YT1 consuming glucose, thus altering the osmotic potential and creating conditions conducive to bacterial growth as well as degradation performance. 

### 3.3. Substrate Utilization Tests

**Figure 6:**
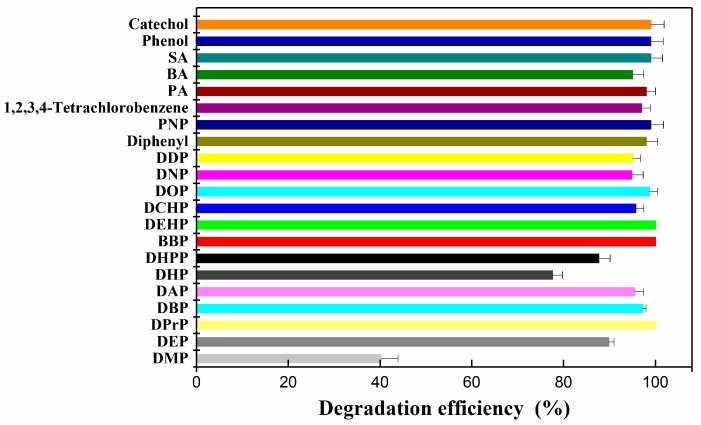
The degradation efficiency of individual phthalic acid esters (PAEs), including DEHP, dimethyl phthalate (DMP), diethyl phthalate (DEP), di-*n*-butyl phthalate (DBP), benzyl butyl phthalate (BBP), di-cyclohexyl phthalate (DCHP), di-propyl phthalate (DPrP), dipentyl phthalate (DAP), dihexyl phthalate (DHP), di-*n*-heptyl phthalate (DHPP), dioctyl phthalate (DOP), di-nonyl phthalate (DNP), and di-decyl phthalate (DDP); and some kinds of aromatic compound, including diphenyl, p-nitrophenol (PNP), 1,2,3,4-tetrachlorobenzene, phthalic acid (PA), salicylic acid (SA), benzoic acid (BA), phenol, and catechol. The structures of the target substrates are presented in Supplementary Figure S1. Error bars indicate standard deviations of the means.

Analyzing the substrate profile was necessary, because in real DEHP-contaminated environments, several kinds of PAEs and organic compounds exist simultaneously [54,55]. The ability of YC-YT1 to degrade many PAEs and organic compounds was investigated at the initial concentration of 100 mg/L and cultivation at 30 °C, 180 rpm, and pH 7.0 for three days. Thirteen typical PAEs (DMP, DEP, DBP, DPrP, DHPP, DNP, DOP, DEHP, DAP, BBP, DDP, DHP, and DCHP) and organic compounds (diphenyl, PNP, PA, BA, phenol, PCA, SA, catechol, and 1,2,3,4-tetrachlorobenzene) were selected for substrate use tests. After three days of incubation on BM supplemented with substrates, 95.5% of DAP, 77.59% of DHP, 87.67% of DHPP, 95.69% of DCHP, 98.7% of DOP, 94.84% of DNP, 95% of DDP, and 100% DEHP and BBP were completely removed (Figure 6). Moreover, YC-YT1 was capable of degrading the major intermediates of PAEs, including PA, BA, SA, phenol, and catechol, as well as diphenyl, PNP, and 1,2,3,4-tetrachlorobenzene.

### 3.4. Maximum and Minimum DEHP Concentration for Efficient Biodegradation

**Figure 7:**
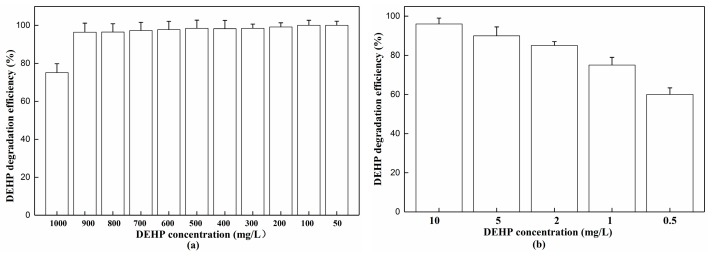
(**a**) Maximum and (**b**) minimum degrading ability of DEHP. The standard error of the mean of the three replicates is represented by the error bar.

Determining the bioremediation application ability against both high and low DEHP concentrations is important. Here, the degradation rates are demonstrated in Figure 7 after three days’ cultivation. Overall, strain YC-YT1 could both survive and remain active in DEHP concentrations as low as 0.5, 1, 2, 5, and 10 mg/L, and as high as 50, 100, 200, 300, 400, 500, 600, 700, 800, 900, and 1000 mg/L. The strain was able to degrade more than 96% of 50–900 mg/L of DEHP. However, when the DEHP concentration was 1000 mg/L, the degradation rate decreased to 75%. Further incubation of strain YC-YT1 in 1000 mg/L for another three days resulted in a degradation rate of 95%. Compared with other DEHP-degraders, strain YC-YT1 is the first that could degrade 60% of 0.5 mg/L DEHP in three days.

### 3.5. DEHP Degradation Pathway

**Figure 8:**
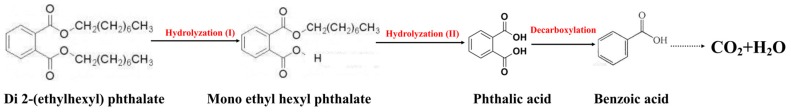
The proposed degradation pathway of DEHP. DEHP was hydrolyzed to phthalic acid (PA) via the intermediate MEHP. BA was metabolized for cell growth through the benzoate metabolism pathway.

Based on HPLC-MS analysis and the corresponding chemical properties (Supplementary Figure S1), the possible degradation pathway of DEHP by *R. ruber* YC-YT1 is outlined in Figure 8. Three metabolites (MEHP, PA, and BA) were detected by HPLC-MS, and the distinct peaks of 277, 165, and 121 *m*/*z* correspond to MEHP, PA, and BA, respectively (Supplementary Figure S3). All three peaks decreased gradually, and none of these compounds were detected after three days. We inferred that strain YC-YT1 might use these compounds as a sole carbon source, which conforms to our findings that no permanent accumulative metabolite was detected at the end of the experiment. In this study, PA was decarboxylated to form BA, which was further exploited for cell proliferation through the benzoic acid degradation pathway.

### 3.6. Bioremediation of DEHP-Contaminated Environments and Evaluation of Cell Surface Hydrophobicity

**Figure 9:**
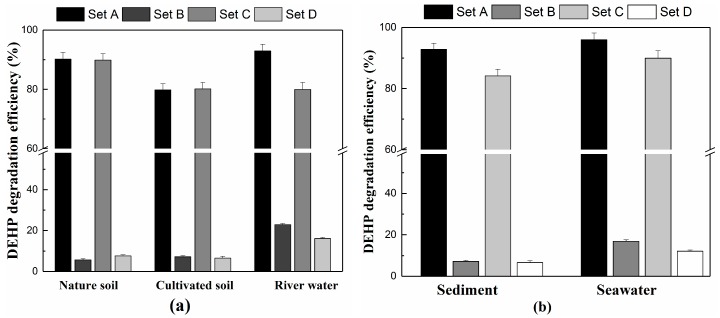
(**a**) The degradation of DEHP by strain YC-YT1 with agricultural soil, garden soil, and river water samples. (**b**) The degradation of DEHP by strain YC-YT1 with sediment and seawater samples. The error bars indicate standard deviations of the means (*n* = 3). The detailed information of sets A, B, C, and D are presented in Table 2.

Several reports [56,57] demonstrated that DEHP appears in agricultural soil and surface water in China. The microbial biomass and basal respiration in soil were inhibited when DEHP concentration reached 100 mg/kg [58]. For this reason, we set the DEHP concentration at 100 mg/kg in samples to measure the efficiency of strain YC-YT1 in degrading DEHP (Supplementary Figure S2). After seven days of incubation, strain YC-YT1 was capable of effectively degrading DEHP in contaminated environments. For the agricultural soil and surface water samples, the total degradation efficiency ranged from 79.7 to 92.9%, whereas the natural degradation efficiency was around 5.6–22.8% (Figure 9a). In addition, strain YC-YT1 showed excellent remediation potential for marine samples. Bioremediation tests in sediment showed that the degradation efficiency of DEHP by strain YC-YT1 ranged from 84.1% to 92.8% within seven days; however, the natural degradation efficiency was maintained at low levels from 6.5% to 7.2%. Furthermore, we found that the seawater bioremediation results by strain YC-YT1 ranged from 89.9–95.9% reduction in the DEHP concentration, and the natural degradation efficiency of DEHP was around 12.8–16.1% (Figure 9b). We hypothesized that the natural degradation of DEHP was very low, and the effects of light depend on the medium component [28]. Light had no impact on the natural degradation rates in garden and wheat field soils or in sediment; however, with the light on, the natural degradation rate in river water and seawater were 1.94 and 1.38 times higher than without light, respectively.

**Figure 10:**
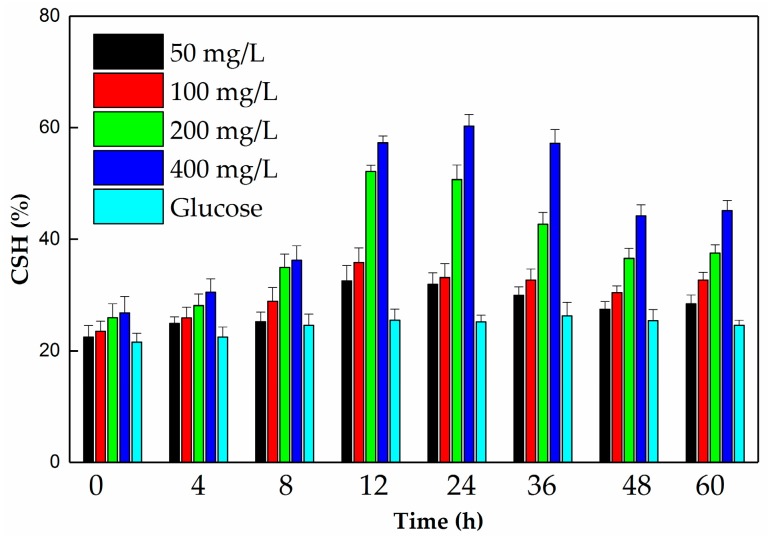
The cell surface hydrophobicity (CSH) of strain YC-YT1 during growth on glucose and different DEHP concentrations. The concentration of glucose was 5 g/L. The error bars indicate the standard deviation of the means (*n* = 3).

Due to the experiments identified, the variation in CSH of strain YC-YT1, the use of DEHP, and the maximum CSH were determined at 12–36 h. However, the variation trends of YC-YT1 among different DEHP concentrations were similar (Figure 10). Compared with glucose, the CSH was higher than when strain YC-YT1 was grown on DEHP. As the DEHP concentration increased, the CSH of YC-YT1 increased. The differences between 50 and 100 mg/L were insignificant. The results agree with the study of Ren [28], which implied that the variation in CSH was related to the use of DEHP.

## 4. Discussion

Many DEHP-degrading isolates have been reported. Bioremediation has the potential to effectively restore the polluted ecosystem based on the biodegradative activities of microorganisms [59]. Among these DEHP-degrading strains, *Rhodococcus* species are a prospective group of bacteria suitable for the biodegradation of persistent pollutants [60]. In fact, they can adjust their cell wall and membrane compositions to survive in contaminated environments [61].

In this research, we isolated an efficient and halotolerant DEHP-degrading strain, *Rhodococcus ruber* YC-YT1. Given the awareness of DEHP and its metabolites’ toxicity, many microorganisms have been isolated from different environments. Table 1 lists several DEHP-degrading bacterial strains that dwell in activated sludge, river sediment, wetlands, etc. However, the pollution due to plastic debris is a serious threat to the oceans [62,63], and high-salinity wastewater of about 5000–6000 mg/L NaCl has been generated in domestic or industrial effluent [22,64,65]. As far as we know, strain YC-YT1 is the first DEHP-degrader isolated from marine plastic debris in costal saline seawater, which could tolerate 0–12% NaCl in BM medium and maintained a DEHP degradation rate above 80% as NaCl concentration increased. High-salinity waste water is an obstacle to effluent treatment, and it affects inherent bacterial enzymatic activity [66]. The osmotic potential of strains with superior salinity tolerance increases, which might affect their metabolic activities [67]. Strain YC-YT1 had good salinity tolerance for bioremediation, which may reduce the cost of desalinization in solid and water waste treatment. Reports have shown that pH and temperature always impact microbial DEHP degradation [10,28,51]. Efficient microbial degradation is generally mediated by enzymes, which prefer to occur under neutral or mildly acidic/alkaline conditions [22,68,69]. However, the ability to degrade contaminants in a wide range of pH and temperature values is indispensable for the isolates. Most DEHP-degraders, such as *Arthrobacter* sp. C21 [39], *Sphingomonas* sp. PA-02 [41], *Acinetobacter* sp. LMB-5 [18], *Gordonia* sp. Dop5 [20], and *Rhodococcus* WJ4 [19], had a high degradation rate at pH 7.0, but could not tolerate high or low pH. Some other studies showed a wide pH range for *Pseudomonas fluorescens* FS1 at 6.5–8.0 [23], *Gordonia alkanivorans* YC-RL2 at 6.0–11.0 [22], *Microbacterium* sp. CQ0110Y at 4.5–9.0 [44], *Acinetobacter* sp. SN13 at 3.0–9.0 [27], and *Rhodococcus* sp. HS-D2 at 5.0–10.0 [31]. Compared with the reported strains, YC-YT1 was able to metabolize DEHP in a wide pH range from 4.0–10.0, and the DEHP degradation rate was about 85% at pH 5.0, whereas it was above 95% at pH 10.0. Strain YC-YT1 also degraded DEHP in a wide range of temperatures, from 10 to 50 °C, with the highest degradation rate at 30 °C. The tolerance to higher temperature of strain YC-YT1 was consistent the *Gordonia* *alkanivorans* YC-RL2 [22] and *Bacillus subtilis* 3C3 [55], which were able to degrade DEHP at temperatures of up to 50 °C. In particular, the DEHP degradation rate of YC-YT1 was above 73% at 10 °C, similar to *Rhodococcus* sp. HS-D2 [31], which was able to metabolize BBP at 15 °C. Strain YC-YT1 has an excellent ability to degrade DEHP in a wide range of pH (4.0–10.0) and temperatures (10–50 °C) and has superior salinity tolerance (0–12% *w*/*v* NaCl). These distinctive characteristics indicate that YC-YT1 may be useful for broader bioremediation applications in contaminated environments.

Given that actual DEHP contamination in the environment is always complex, several kinds of PAEs and polychlorinated biphenyls (PCBs) exist simultaneously [54]. Hence, it was necessary to analyze the substrate profile. In most cases to date, many strains have been reported that could use several kinds of PAEs. For instance, *Pseudomonas fluorescens* FS1 [23], *Sphingomonas* sp. PA-02 [41], *Acinetobacter* sp. LMB-5 [18], *Agromyces* sp. MT-O [13], and *Mycobacterium* sp. YC-RL4 [28] could use no more than five kinds of PAEs. In addition, for the reported biodegradation process of PAEs (including DEHP), PA is the central intermediate and transforms first into another key intermediate [10,70] which is a recalcitrant compound that is suspected to cause cancer and kidney damage [11,71]. Indeed, not all bacteria can transform DEHP into certain intermediates like PA or BA. For example, *Gordonia* sp. Dop5 failed to use PA [20]. Conversely, *Rhodococcus* sp. HS-D2 [31], *Bacillus subtilis* No.66 [21], *Gordonia* *alkanivorans* YC-RL2 [22], *Providencia* sp. 2D [11], and *Arthrobacter* C21 [39] completely degraded PAEs. Compared with the reported strains, *Rhodococcus ruber* YC-YT1 is capable of efficiently metabolizing all of the listed chemicals in the text. Thus, this study is the first report of a bacterium that can simultaneously degrade 13 kinds of PAEs as well as polychlorinated biphenyl. Moreover, long-term exposure to lower concentrations of DEHP is a serious threat to human health [12]. Environments with lower concentrations pose difficulties for biodegradation because the concentration of pollutants is so low that they do not support bacterial growth or induce the expression of functional genes [10,51]. However, determining the bioremediation application ability against high-concentration pollutants is also necessary [22,51]. Therefore, more investigations into YC-YT1 for various DEHP concentration ranges should be completed. In this study, YC-YT1 could both survive and remain active in DEHP concentrations as low as 0.5 mg/L and as high as 1000 mg/L. Compared with other DEHP degraders, strain YC-YT1 has an excellent ability to metabolize high DEHP concentrations [20,22,27,31,42]. Importantly, YC-YT1 is also the first report that could degrade 60% of 0.5 mg/L DEHP in three days. However, further investigation is needed to determine the mechanism of DEHP biodegradation.

The metabolic pathway of DEHP can be divided into two processes: DEHP completely de-esterifies to form PA and use of PA [70]. The analysis of intermediates indicated that MEHP, BA, and PA exist during degradation by *Rhodococcus ruber* YC-YT1. The degradation pathway involving strain YC-YT1 was proposed, which is similar to the strains of *Gordonia* *alkanivorans* YC-RL2 [22], *Pseudomonas fluorescens* FS1 [23], and *Microbacterium* sp. CQ0110Y [44]. Firstly, DEHP was observed to hydrolyze two ester bonds sequentially and generate PA. Then, PA was decarboxylated to BA, which is the central intermediate in anaerobic phthalate metabolism [70]. BA was used via ring hydroxylation or β-oxidation to form phenol and catechol, which were later metabolized in the tricarboxylic acid (TCA) cycle [30]. Because BA is an intermediate of DEHP degradation by strain YC-YT1, we assumed that the strain has functional genes involved in PA catabolism. Strain YC-YT1 was also able to completely degrade PA, phenol, and catechol as sole carbon sources in three days. In previous studies of DEHP biodegradation, considerable progress has been made in the bioremediation of DEHP-contaminated environments. Compared with other methods, bioremediation can irreversibly depolymerize the composition the pollutants [72,73]. However, the available DEHP-degrading strains are limited. Wang determined that *Rhodococcus* sp. WJ4 was able to completely degrade 200 mg/L DEHP in soil within seven days and the microbiological function of the DEHP-contaminated soil was restored [19]. Xu reported a new method of improving DEHP-contaminated soils by combining biochar with compost manure, with 55.5–69.8% of 100 mg/L DEHP decomposing at the end of the experiment [27]. Bioremediation of the wetlands contaminated with phthalates was performed with *Arthrobacter* sp. C21, which is a DBP-degrader [39]. Previous studies have revealed that bacteria with high hydrophobicity could enhance the adherence to pollutant surfaces, which could overcome the bioavailability limitation of these contaminants, and hence accelerate biodegradation [28,74,75]. For example, *Mycobacterium* sp. YC-RL4 [28], a DEHP-degrading strain, can adjust its CSH to enhance the bioavailability of DEHP. The total removal efficiency for this strain ranged from 64.7% to 87.1%. In this study, we determined that the CSH of strain YC-YT1 when grown on DEHP was apparently higher than on glucose. As the DEHP concentration increased, the CSH of YC-YT1 increased, though the differences between 50 and 100 mg/L were insignificant. Maximum CSH appeared between 12 and 36 h when using DEHP for the growth of strain YC-YT1. This result implies that the variation in CSH is connected to the degradation of DEHP, which agrees with the study of Ren [28]. Strain YC-YT1 also effectively metabolized 100 mg/kg DEHP in contaminated agricultural soils, coastal sediment, pond water, and coastal seawater within seven days, with a total degradation efficiency of around 79.7–95.9%, which was superior to that of *Mycobacterium* sp. YC-RL4 [28]. However, the natural degradation efficiency was around 5.6–22.8% with the light on, and the natural degradation rate in pond water and coastal seawater was respectively 1.94 times and 1.38 times higher than that without light. Compared with the agricultural soil and river water, the marine sample degradation efficiency of strain YC-YT1 was just slightly higher. This study is the first report about bioremediating DEHP-contaminated marine samples. Moreover, Ren hypothesized that light-mediated degradation should be an important process in water [28], and some indigenous microbial degradation also occurs [76,77]. Based on these results, we inferred that strain YC-YT1 was able to adapt to the environment by modulating its cell surface hydrophobicity. Apart from the components of the cell outer membrane, the mechanism responsible for CSH variation is unclear, and will be the subject of our future work. Although bioremediating DEHP-contaminated environments has not yet been broadly explored, the bioprocess with various environmental samples has demonstrated its application potential. 

## 5. Conclusions

A highly-efficient DEHP-degrading bacterium with halotolerance named YC-YT1 was isolated from marine plastic debris in coastal saline seawater and identified as *Rhodococcus ruber* by 16S rRNA gene analysis and BIOLOG tests. The strain completely degraded DEHP (100 mg/L) at pH 7.0 and temperature 30 °C within three days. Strain YC-YT1 could tolerate NaCl concentrations of up to 12% and possessed the broadest substrate spectrum for DEHP-degradation bacteria from genus *Rhodococcus*. The maximum and minimum initial concentrations of DEHP ranged from 0.5 to 1000 mg/L and the degradation efficiency and CSH when grown on DEHP were evaluated. Using HPLC-MS analysis, the DEHP intermediates were detected and the degradation pathway was deduced. To the best of our knowledge, this study is the first report about a halotolerant *Rhodococcus ruber* that can simultaneously degrade 13 kinds of PAEs and diphenyl, PNP, PA, BA, phenol, PCA, SA, catechol, and 1,2,3,4-tetrachlorobenzene. Furthermore, DEHP-contaminated soil and water were remarkably remedied by the strain YC-YT1, and the degradation rates were approximately 79.7–95.9%. These results prove that strain YC-YT1 exhibits outstanding application potential for the bioremediation of DEHP-contaminated environments.

## Figures and Tables

**Table 1:** Typical di-(2-ethylhexyl) phthalate (DEHP)-degrading bacteria strains and their characteristics.

Sources	Strain	Experimental Conditions	Substrate (Substrate Profile)	Degradation Efficiency	Major Intermediates	References
Marine plastic debris in coastal seawater	*Rhodococcus ruber* YC-YT1	pH 7.0, 30 °C (pH 4–10.0, Temperature 10–50 °C)	DEHP (DDP, DNP, DOP, DCHP, BBP, DHPP, DHP, DAP, DBP, DPrP, DEP, DMP, PA, BA, SA, PNP, diphenyl, 1,2,3-tetrachlorobenzene, PCA, catechol)	100 mg/L, 100% DEHP, 3 days	MEHP, PA, BA	**This study**
Activated sludge	*Pseudomonas fluorescens* FS1	pH 6.5–8.0, 30 °C Inhibition below 10 °C and above 35 °C)	PAEs (DMP, DEP, DnBP, DIBP, DnOP, DEHP)	100 mg/L, 99% DMP, DEP, DnBP, DIBP; 20% DnOP, 30% DEHP, 3 days	NR	[23]
River sediment and petrochemical sludge	*Sphigomonas* sp. DK4 cooperation with *Corynebacterium* sp. O18	pH 7.0, 30 °C (pH 5–9, Temperature 20–40 °C)	DEP, DPrP, DBP, DPP, DCP, DHP, BBP, DEHP	5 mg/L, 32.6% DEHP and 91.6% DEHP, respectively, 7 days	NR	[24]
River sediment	Anaerobic bacteria community	pH 7.0, 30 °C (pH 5–9, 20–50 °C)	DEP, DBP, DEHP	5 μg/g, 91% DEP, 94.1% DBP, 95.4% DEHP, 84 days	NR	[38]
Constructed wetland soil	*Arthrobacter* C21	pH 7.0, 30 °C	DBP (DMP, DEP, DOP, DEHP, PA)	100 mg/L, 51.4%, DEHP, 70 h	NR	[39]
Heavily plastics-contaminated sewage sludge	*Achromobacter denitrificans* SP1	pH 8.0, 32 °C	DEHP	10 mM, 63% DEHP, 72 h; 100% DEHP 96 h	MEHP, 2-ethyl hexanol	[40]
Aerobic granules	*Sphingomonas* sp. PA-02	pH 7.0, 25–35 °C	DMP (DBP, DEP, DEHP)	29–33% 290 mg/L DMP, 5 days	PA, BA	[41]
Wetland soil	*Bacillus megaterium* YJB3	34.2 °C	DBP (PA, PCA, MBP, DMP, DEP, DBP, DEHP, DnOP, DINP)	1000 mg/L, 82.5% DBP, 5 days	MBP, PA, PCA	[2]
Compost-amended soil	*Providencia* sp. 2 D	NR	DMP (DEP, DBP, DnOP, DEHP, MBP, PA, BA, PCA, Catechol)	200 mg/L, 100% DBP, 3 days	MBP, PA	[11]
Activated sludge	Bacterial community	pH 7.0, 30 °C	DEP (DBP, BBP, DEHP)	50 mg/kg, 95.2% DEHP, 98.6% BBP, 99.2% DBP, 98% DEP	NR	[42]
Vegetable greenhouse soil	*Acinetobacter* sp. LMB-5	pH 7.0, 40 °C	DMP (DEP, DBP, DEHP)	100 mg/L, 100% DBP, 60 h, 98.87% DMP, 94.94% DEP, 72.15% DBP, 45 h	DMP, PA	[18]
Organic amendment soil	Bacterial community	NR	DBP, DEHP	NR	NR	[43]
Municipal waste	*Gordonia* sp. Dop5	pH 7.0, 28 °C	DMP, DEP, DnBP, DnOP, DEHP, BBP, DPP, MnOP, PCA, no PA	750 mg/L, 100% DnOP 48 h	CO_2_, H_2_O	[20]
Petroleum-contaminated soil	*Gordonia* *alkanivorans* YC-RL2	pH 8.0, 30 °C, 0–5% NaCl (pH 6–11, Temperature 10–50 °C, NaCl 0–12%)	DEHP, DBP, DCHP, DMP, DEP, PA	800 mg/L, 94.6% DEHP, 7 days	MEHP, PA, BA	[22]
Activated sludge	*Microbacterium* sp. CQ0110Y	pH 6.5–7.5, 25–35 °C (pH 4.5–9.0, Temperature 10–50 °C, 10 °C and 50 °C no degradation)	DEHP	1000 mg/L, 100% DEHP, 10 days	MEHP, PA, BA, PCA, muconic acid, pyruvic acid	[44]
Activated sludge	*Acinetobacter* sp. SN13	pH 6–9, 30 °C, (pH 3–9, Temperature 25 °C, 30 °C, 35 °C)	DEHP	400 mg/L, 90% DEHP, add 100–1000 μg/L Fe^3+^ can improve degradation rate, 100 μg/L Mn^2+^ can improve but 500–1000 μg/L Mn^2+^ inhibition	MEHP, PA, PCA	[27]
Vegetable soil	*Rhodococcus* WJ4	pH 7.0, 28 °C	DEHP	200 mg/L, 96.4% DEHP, 7 days	NR	[19]
Soil	*Bacillus subtilis* No. 66	pH 7.5, 30 °C	DEHP (DBP, DEP, DPP, DPrP, PA)	5 mM, 99% DEHP, 5 days	MEHP, PA, PCA	[21]
Soil	*Bacterial community*, *G1, Rhodococcus rhodochrous* G2, *Rhodococcus rhodochrous* G7, *Corynebacterium nitrilophilus* G11	pH 7.0, 30 °C	DEHP	100 mg/L, G1, G2, 97%DEHP, 3 days; G7, 32.5% DEHP, G11 cooperation with surfactant 90% DEHP within 24 h	NR	[45]
Garden soil	*Mycobacterium* sp. NK0301	pH 6.8, 30 °C	DEHP	98% 0.1% (*v*/*v*) DEHP within 24 h	2-ethylhexanol, 1,2-benzenedicarboxylic acid	[46]
Contaminated river sediment	*Rhodococcus* sp. HS-D2	pH 7.0 30 °C (pH 5–10, Temperature 15–42 °C)	BBP (DMP, DEP, DBP, DOP, DEHP, PA, catechol, pyridine, BA, Tween-80)	500 mg/L, 100% BBP, 96 h	MBP, PA, BA	[31]
NR	*Agromyces* sp. MT-O	pH 7.2, 29.6 °C	DEHP (DMP, DEP, DBP, DOP)	200 mg/L, 90% DEHP, 4 days	MEHP, PA	[13]
Soil	*Mycobacterium* sp. YC-RL4	pH 8.0, 30 °C	DEHP (DCHP, DBP, DEP, DMP)	50 mg/L, 100% DEHP, 5 days	MEHP, PA	[28]
Mixed pulper waste	*Fusarium culmorum*	pH 6.5, 28 °C	DEHP	1000 mg/L, 95% DEHP, 60 h	MEHP, PA, PCA, butanediol	[47]
Purchased	*Pleurotus ostreatus*	25 °C	DEHP	1000 mg/L, 100% DEHP, 21 days	MEHP, 2-ethyl-hexan-1-ol, PA	[1]

NR: not reported; dimethyl phthalate (DMP), diethyl phthalate (DEP), di-*n*-butyl phthalate (DBP), benzyl butyl phthalate (BBP), di-cyclohexyl phthalate (DCHP), di-propyl phthalate (DPrP), dipentyl phthalate (DAP), dihexyl phthalate (DHP), di-*n*-heptyl phthalate (DHPP), dioctyl phthalate (DOP), di-nonyl phthalate (DNP), and di-decyl phthalate (DDP); di-*n*-butyl phthalate(DnBP); diiso-butyl ortho-phthalate (DIBP); dioctyl phthalate (DnOP); diisononyl ortho-phthalate (DINP); mono (2-ethylehxyl) phthalate (MEHP); mono-*n*-octyl phthalate (MnOP); phthalic acid (PA); phthalic acid ester (PAEs); protocatechuic acid (PCA); benzoic acid (BA); mono-*n*-butyl phthalate (MBP).

**Table 2:** Experimental sets for DEHP biodegradation with environmental samples.

Sets		Inoculum Volume (mL of Seeds)	DEHP Concentration (mg/kg or mg/L)	Photoperiod (Light: Dark)	Light Intensity (lx)
1	A	3	100	16:8	5 × 10^3^ 5 × 10^3^ - -
	B	0	100	16:8	
2	C	3	100	-	
	D	0	100	-	

## Supplementary Materials

The following are available online at http://www.mdpi.com/1660-4601/15/5/964/s1. Figure S1: The structures of target contaminants (the name was listed under the structure). Figure S2: Partial photos of the bioprocess with environmental samples. (A, natural soils with light after 5 days incubation; B, river water with light after 5 days incubation; C, Sediments with light after 5 days incubation; D,: Seawater with light after 5 days incubation.) Sterilized water was supplemented when necessary. Figure S3: HPLC-MS analysis results of DEHP degradation intermediates. The chemical structure of potential intermediates was presented in each figure., Figure. S4: The sediment samples in intertidal zone from the Dameisha coastline. Table S1: Physicochemical properties of soils. Table S2: Physicochemical properties of water. Table S3: Physicochemical properties of coastal sediment and seawater.
