# Supplementary material for: Biodegradation of Di-(2-ethylhexyl) Phthalate by Rhodococcus ruber YC-YT1 in Contaminated Water and Soil
Source: Int J Environ Res Public Health. 2018 May 11;15(5):964. doi: 10.3390/ijerph15050964 (PMC5982003; doi:10.3390/ijerph15050964)
Supplement: Supplementary file 1 [file ijerph-15-00964-s001.pdf]

Biodegradation of Di-(2-ethylhexyl) phthalate by *Rhodococcus ruber* YC-YT1 in Contaminated Water and Soil

Ting Yang [1], Lei Ren [2], Yang Jia[1], Shuanghu Fan[1], Junhuan Wang[1], Jiayi Wang[1], Ruth Nahurira[1], Haisheng Wang[1], and Yanchun Yan [1,*]

[1] Graduate School, Chinese Academy of Agricultural Sciences, Beijing 100081, China; e-mail 13838348327@163l.com
[2] Agricultural College of Guangdong Ocean University, Zhanjiang 524088, China; e-mail lren_87@hotmail.com
* Correspondence: e-mail yanyanchun@caas.cn; Tel.: +86 10 82109685

Figure. S1. The structures of target contaminants (the name was listed under the structure).

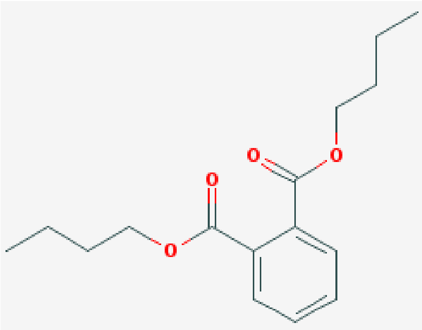

Dibutyl phthalate (DBP)

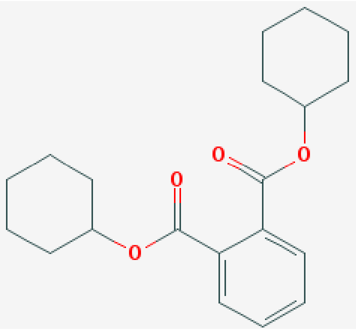

Dicyclohexyl phthalate (DCHP)

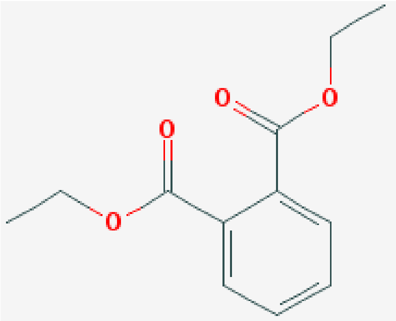

Diethyl phthalate (DEP)

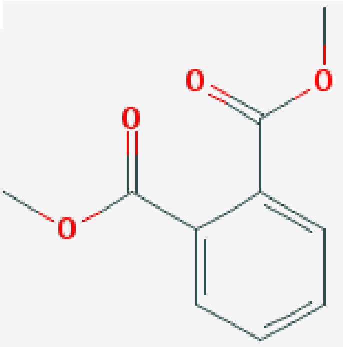

Dimethyl phthalate (DMP)

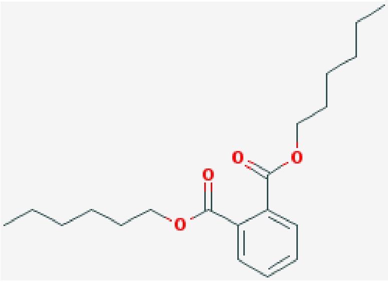

Dihexyl phthalate (DHP)

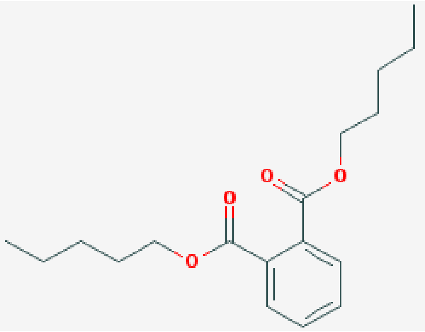

Dipentyl phthalate(DAP)

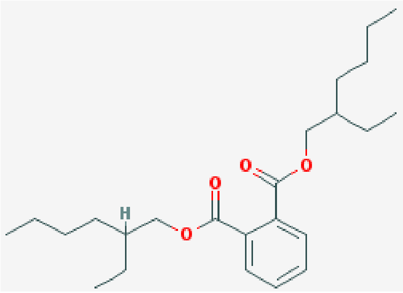

Di-(2-ethyl hexyl) phthalate (DEHP)

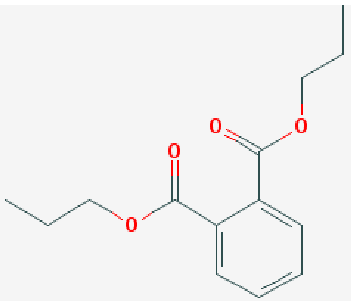

Dipropyl phthalate (DPrP)

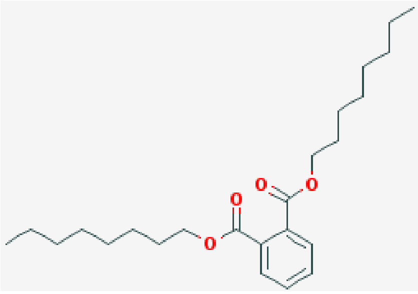

Di-n-octyl phthalate (DOP)

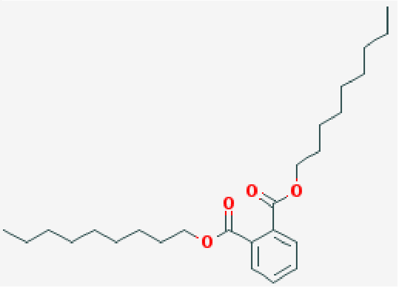

Dinonyl phthalate (DNP)

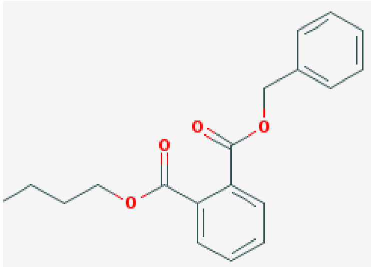

Butyl benzyl phthalate (BBP)

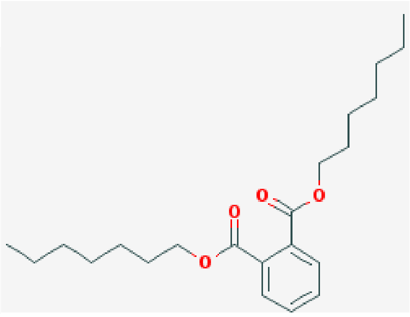

Di-n-heptyl phthalate (DHPP)

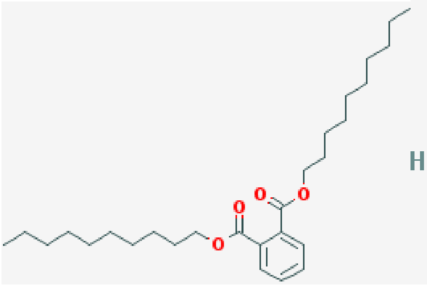

Didecyl phthalate (DDP)

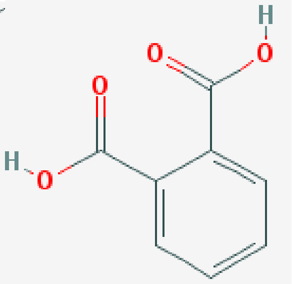

Phthalate acid (PA)

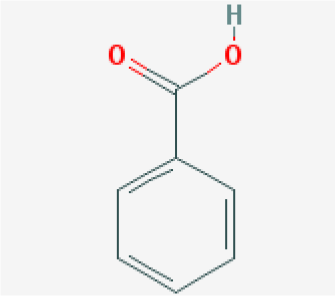

Benzoic acid (BA)

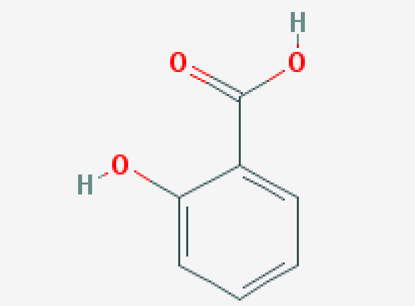

Salicylic acid (SA)

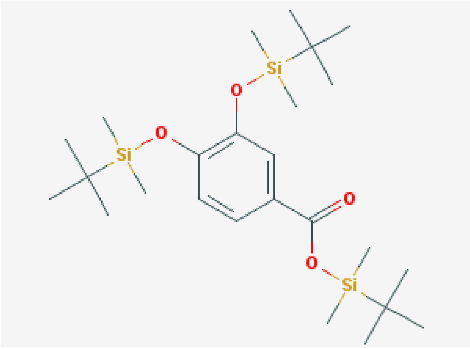

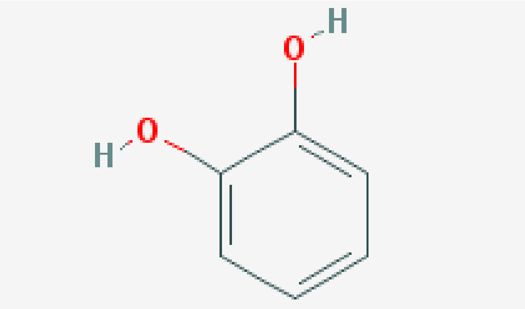

Protocatechuic acid (PCA) Catechol

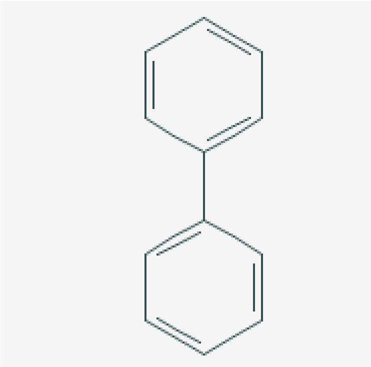

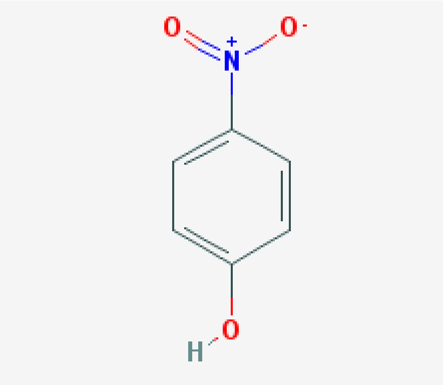

Diphenyl p-nitrophenol (PNP)

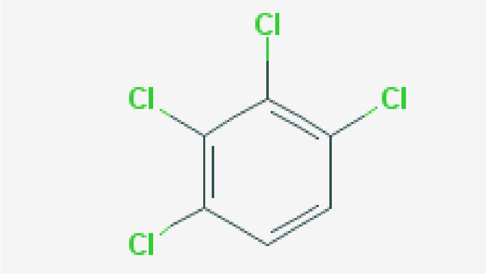

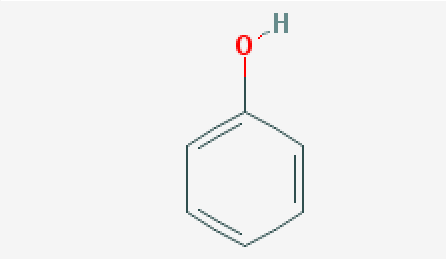

1,2,3,4-tetrachlorobenzene Phenol

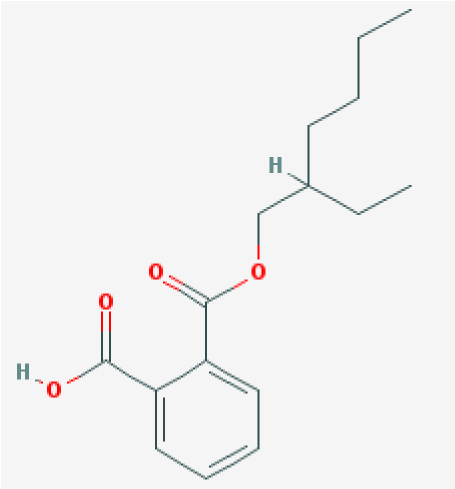

Mono (2-ethylehxyl) phthalate (MEHP)

Figure. S2. Partial photos of the bioprocess with environmental samples. (A,natural soils with light after 5 days incubation; B, river water with light after 5 days incubation; C, Sediments with light after 5 days incubation; D,: Seawater with light after 5 days incubation.) Sterilized water was supplemented when necessary.

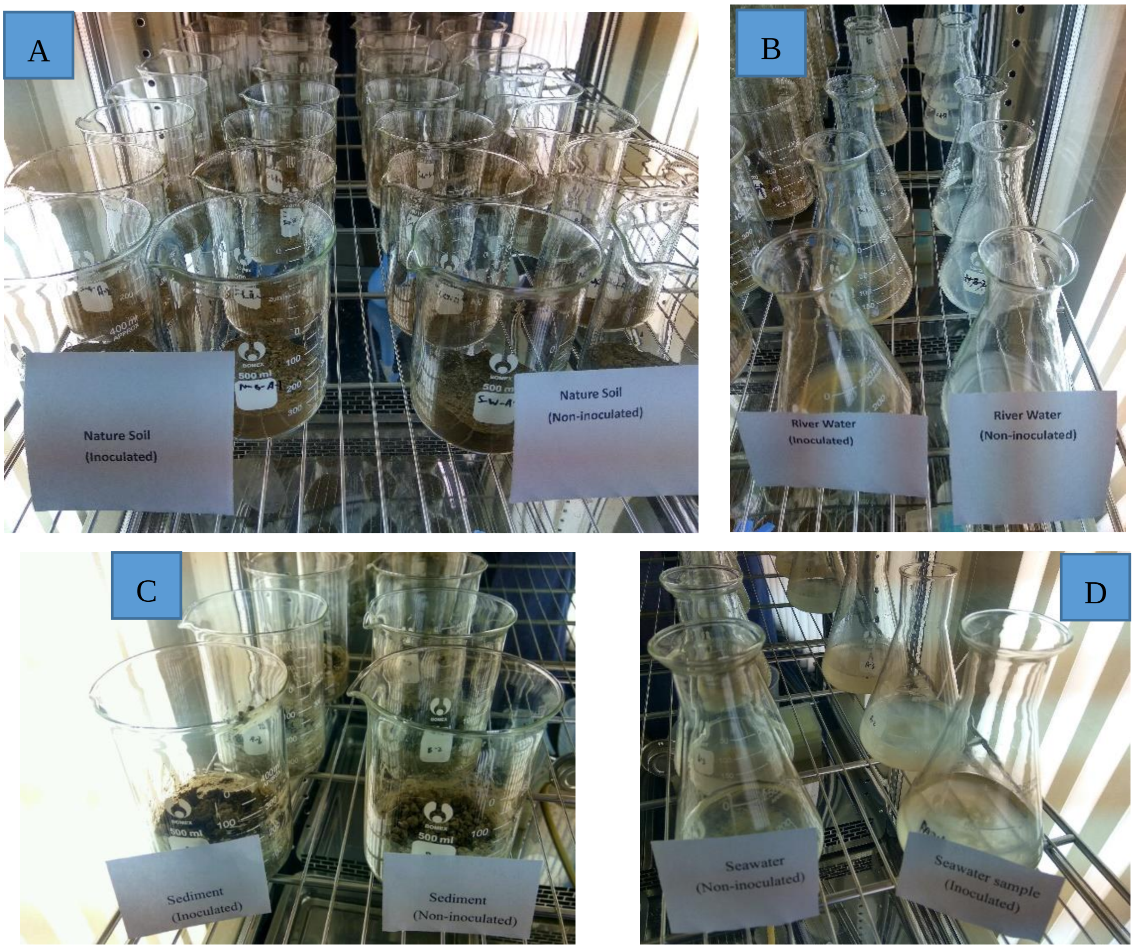

Figure. S3. HPLC-MS analysis results of DEHP degradation intermediates. The chemical structure of potential intermediates was presented in each figure.

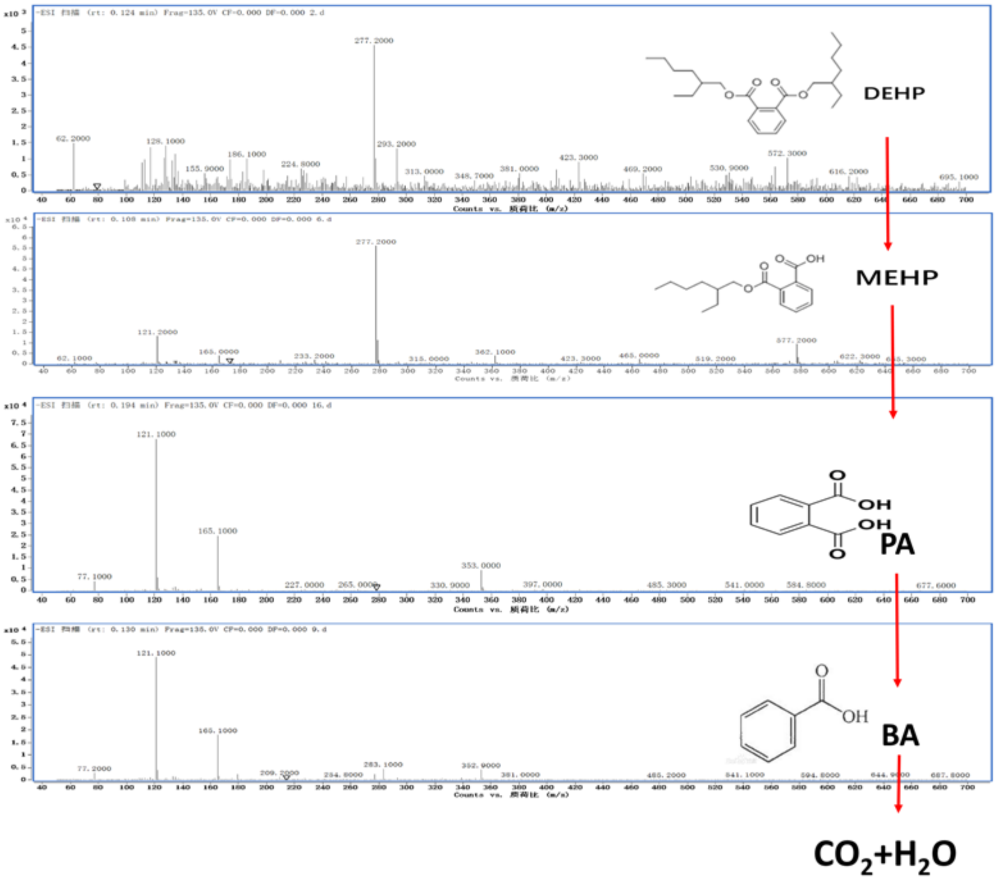

Figure. S4. The sediment samples in intertidal zone from the Dameisha coastline.

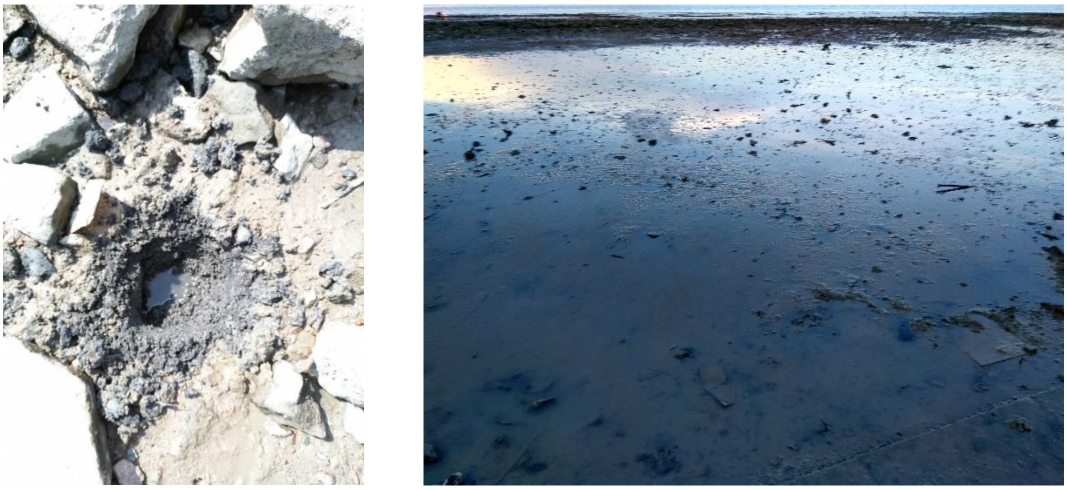

Table S1. Physicochemical properties of soils

Soil type	Source	Location	pH[1]	OM (g/kg)	TN (g/kg)	TP (g/kg)	DEHP concentration (mg/kg)
Cultivated	Wheat field	45°40'N, 126°35′E	6.39	27.26	1.20	0.22	0.31
Natural	Garden Soil	39°57'N, 116°19′E	7.23	37.27	1.37	0.14	ND

OM means organic matter. TN means total nitrogen. TP means total phosphorus. DEHP means di-(2-ethyl hexyl) phthalate. ND means not detected.

Table S2. Physicochemical properties of water.

Water type	Location	pH[1]	SS (mg/kg)	COD (mg/L)	Conductivity (μS cm^{-1})	DEHP concentration (mg/L)
River water	40°02'N, 126°29′E	7.42	12.37	23.7	103.9	0.24

SS means suspended solid. COD means chemical oxygen demand and it was determined by the potassium chromate method. DEHP means di-(2-ethyl hexyl) phthalate.

Table S3. Physicochemical properties of coastal sediment and seawater.

Sediment		Seawater	
Location	22°59'N, 114°31′E	Location	22°59'N, 114°31′E
pH[1]	7.7	pH[1]	7.6
Salinity(%)	3.22	Salinity(%)	3.61
ORP (mV)	-240	ORP (mV)	50
Moisture content (%)	58.8	Optical density	0.86
TOC (ng/g)	7905	Conductivity (μS cm^{-1})	523
TN (g/kg)	2.2	SS (mg/kg)	15.62
TP (g/kg)	1.3	COD (mg/L)	25.1
DEHP concentration (mg/kg)	7.1	DEHP concentration (mg/L)	3.8

TN means total nitrogen. TP means total phosphorus. ORP means oxidation-reduction potential. TOC means total organic carbon. SS means suspended solid. COD means chemical oxygen demand and it was determined by the potassium chromate method. DEHP means di-(2-ethyl hexyl) phthalate.
